# Site-directed mutagenesis of *Mycobacterium tuberculosis* and functional validation to investigate potential bedaquiline resistance-causing mutations

**DOI:** 10.1038/s41598-023-35563-0

**Published:** 2023-06-06

**Authors:** Christian C. Otum, Emmanuel Rivière, Monique Barnard, Johannes Loubser, Monique J. Williams, Elizabeth M. Streicher, Annelies Van Rie, Robin M. Warren, Marisa Klopper

**Affiliations:** 1 Tuberculosis Omics Research Consortium, Family Medicine and Population Health, Institute of Global Health, Faculty of Medicine and Health Sciences, University of Antwerp, Antwerp, Belgium; 2 Department of Science and Innovation (DSI)-National Research Foundation (NRF) Centre of Excellence for Biomedical Tuberculosis Research, South African Medical Research Council Centre for Tuberculosis Research, Division of Molecular Biology and Human Genetics, Faculty of Medicine and Health Sciences, Stellenbosch University, Cape Town, South Africa; 3 Department of Molecular and Cell Biology, University of Cape Town, Cape Town, South Africa

**Keywords:** Biological techniques, Computational biology and bioinformatics, Microbiology, Molecular biology, Diseases, Infectious diseases, Tuberculosis

## Abstract

Molecular detection of bedaquiline resistant tuberculosis is challenging as only a small proportion of mutations in candidate bedaquiline resistance genes have been statistically associated with phenotypic resistance. We introduced two mutations, *atpE* Ile66Val and *Rv0678* Thr33Ala, in the *Mycobacterium tuberculosis* H37Rv reference strain using homologous recombineering or recombination to investigate the phenotypic effect of these mutations. The genotype of the resulting strains was confirmed by Sanger- and whole genome sequencing, and bedaquiline susceptibility was assessed by minimal inhibitory concentration (MIC) assays. The impact of the mutations on protein stability and interactions was predicted using mutation Cutoff Scanning Matrix (mCSM) tools. The *atpE* Ile66Val mutation did not elevate the MIC above the critical concentration (MIC 0.25–0.5 µg/ml), while the MIC of the *Rv0678* Thr33Ala mutant strains (> 1.0 µg/ml) classifies the strain as resistant, confirming clinical findings. In silico analyses confirmed that the *atpE* Ile66Val mutation minimally disrupts the bedaquiline-ATP synthase interaction, while the *Rv0678* Thr33Ala mutation substantially affects the DNA binding affinity of the MmpR transcriptional repressor. Based on a combination of wet-lab and computational methods, our results suggest that the *Rv0678* Thr33Ala mutation confers resistance to BDQ, while the *atpE* Ile66Val mutation does not, but definite proof can only be provided by complementation studies given the presence of secondary mutations.

## Introduction

In 2012, bedaquiline (BDQ) became the first new drug administered for the treatment of tuberculosis (TB) in more than forty years^1^. Since 2018, BDQ forms part of the three core drugs used for the treatment of rifampicin resistant tuberculosis (RR-TB)^2^. Unfortunately, BDQ treatment failure due to BDQ-resistant TB has already emerged, threatening the gains made in treatment outcomes for RR-TB^3–6^.

BDQ resistance is caused by mutations in the tier 1 (*atpE*, *Rv0678*, *Rv0676c*, *Rv0677c*, and *pepQ*) and tier 2 (*Rv1979c*) candidate resistance genes of *Mycobacterium tuberculosis* (*Mtb*)^7^. Mutations in the *Rv0678* gene, encoding MmpR, a transcriptional repressor of the MmpL5/MmpS5 efflux pump system, are considered to be the main drivers of clinical BDQ resistance^3^. Mutations in the *atpE* gene, encoding the essential BDQ drug target ATP synthase subunit C, greatly impact the BDQ phenotype in vitro, but are rarely observed in clinical isolates^6,8^. In a recent study by the WHO of over 38,000 samples, 537 different mutations in BDQ resistance candidate genes were observed but only four (0.7%) were significantly associated with BDQ susceptibility and none were associated with BDQ resistance. The other 533 (99.3%) mutations were graded as “uncertain significance” due to insufficiency of data^7^. A similar observation was made in a recent systematic review, where only two (0.7%) of 290 mutations could be statistically associated with BDQ resistance^8^. The insufficiency of data highlighted by these studies directly impacts the potential accuracy and development of molecular diagnostics to detect BDQ resistance^9^.

Alternative methods to determine the molecular mechanisms of BDQ resistance are needed. In vitro mutagenesis through exposure to low concentrations of BDQ has shown interesting results, where *Rv0678* mutations appear to be transient, conferring low-level resistance, while potentially leading to acquisition of additional, high level resistance conferring mutations in the *atpE* gene^10^. However, the emerging mutation profiles are highly variable and unpredictable and cannot be used to study specific genomic mutations. Site-directed mutagenesis can be used to determine the phenotype associated with specific mutations in candidate resistance genes. In this study, we introduced one point mutation in the *atpE* gene [c.196A>G (Ile66Val)] and one point mutation in the *Rv0678* gene [c.97A>G (Thr33Ala)] of an *Mtb* H37Rv reference strain, to study the effects of these different point mutations on the minimal inhibitory concentrations (MIC) for BDQ. These mutations were selected for mutagenesis in *Mtb* based on recent reports in literature^3,11^. The *atpE* Ile66Val mutation has been reported to occur in a phenotypically susceptible clinical isolate (BDQ MIC of 0.125 µg/ml on the resazurin microtiter plate assay [REMA])^11^, which is atypically low for *atpE* mutations as these usually confer high level BDQ resistance. The *Rv0678* Thr33Ala mutation was selected because it has been observed in in vitro isolates with very high MIC values (8 µg/ml on the mycobacterial growth indicator tube [MGIT] platform and > 2 µg/ml on 7H10 medium), both in combination with other mutations and as a single mutation^10,12^. This is rather unusual as *Rv0678* mutations tend to confer a low level of BDQ resistance^8^. We explored two different techniques to achieve this goal, namely homologous recombineering and homologous recombination, to expand our repertoire of mutagenesis techniques.

## Methods

We aimed to introduce clinically relevant mutations (*atpE* Ile66Val and *Rv0678* Thr33Ala) that have been associated with BDQ resistance into a known genetic background using site-directed mutagenesis, to establish their influence on BDQ MIC. We further aimed to support our findings by demonstrating the effect of the mutations on the relevant protein structures and BDQ binding affinity through in silico modelling.

### Mutation selection for site directed mutagenesis

Two mutations were selected for mutagenesis and functional validation in *Mtb* H37Rv, namely the c.196A>G (p.Ile66Val) mutation in the *atpE* gene and the c.97A>G (p.Thr33Ala) mutation in the *Rv0678* gene.

FastQ DNA sequence data from Mycobrowser and NCBI were utilized to design a 63 bp long single stranded DNA (ssDNA) oligonucleotide for recombineering, carrying a single nucleotide base pair change of interest (*atpE* c.196A>G [p.Ile66Val]) centrally (Supplement Table S1.1). Similarly, 41 bp long overlapping DNA primers were designed for recombination to amplify *Mtb* wild-type DNA and incorporate the *Rv0678* c.97A>G (p.Thr33Ala) mutation in a newly synthesised double stranded DNA (dsDNA) fragment (Supplement Table S2.1). Oligonucleotides, purified at 100 nm scale with polyacrylamide gel electrophoresis (PAGE), were acquired from Integrated DNA Technologies.

### Bacterial strains and culture conditions

*Escherichia coli* (*E. coli*) strains XL1-blue and DH5α were used for plasmid propagation and cloning. *E. coli* was cultured in Lysogeny broth (LB) or on LB agar at 37 °C. When appropriate, ampicillin (AMP; 100 μg/ml), kanamycin (KAN; 25 μg/ml), 5-Bromo-4-Chloro-3-Indolyl β-D-Galactopyranoside (X-gal) (50 μg/ml), hygromycin (HYG; 50 μg/ml) or sucrose (2%) was added for selection. The *Mtb* ATCC® 27294 (TMC 102) H37Rv strain was used for site-directed mutagenesis^13^. *Mtb* was grown in Middlebrook 7H9 broth and (unless otherwise stated) supplemented with 0.05% Tween-80, 0.2% glycerol, and 10% oleic acid-albumin-dextrose-catalase (OADC) (7H9|GLY|OADC|Tween-80). The plasmid vectors p2NIL and pGOAL19 were used for the homologous recombination method as developed by Parish and Stoker^14^ (Supplement Fig. S2).

### Homologous recombineering

The episomal plasmid vector pJV75amber, expressing recombinase protein gp61 (encoded by *Che9c*), was acquired from the van Kessel laboratory (Department of Biology, Indiana University), and was used for homologous recombineering according to the method of van Kessel and Hatful^15,16^ (Supplement Fig. S1). A log phase culture of *Mtb* (pJV75amber) was induced with acetamide solution (0.2%) for 24 h to increase the expression of gp61 recombinase protein.

Following induction, the cells were transformed with 500 ng of recombineering ssDNA oligonucleotide carrying the mutation of interest and 100 ng JCV198. A transformation with 500 ng of recombineering ssDNA oligonucleotide only was included as a control for recombineering efficiency. Following recovery, transformation mixtures were cultured on agar plates with and without HYG. HYG resistant colonies were inoculated in 7H9|GLY|OADC supplemented medium and incubated at 37 °C for 21 days. Subsequently, heat-killed cultures were centrifuged at 3500 rpm and the supernatant was used for PCR amplification (Supplement Table S1.2) with in-house designed primer sets (Supplement Table S1.1). Sanger sequencing of PCR products was performed to confirm the presence of the *atpE* mutation of interest.

### Homologous recombination

A homologous region of 2825 bp containing the *Rv0678* c.97A>G (p.Thr33Ala) mutation in the middle was generated by overlap-PCR amplification from *Mtb* H37Rv genomic DNA (Supplement Recombination), whereby the primers are designed to introduce the mutation in the centre of the fragment (Supplement Tables S2.1–S2.5). Sanger sequencing was used to confirm that the desired mutation was introduced (Supplement Tables S2.1 and S2.2). The fragment was cloned into pJET1.2 to sequence the DNA fragment, then excised and cloned into the HindIII and KpnI sites in p2NIL, and selection and counter-selection markers from pGOAL19 (Supplement Table S2.6) were added using a unique PacI restriction enzyme site to generate the allelic exchange substrate^17^ (Supplement Fig. S2). The original pJET1.2, p2NIL and pGOAL19 plasmids are available from Addgene, while the altered versions are available upon request from the authors.

Electrocompetent *Mtb* cells were transformed with the allelic exchange substrate carrying the mutated *Rv0678* fragment and selected on 7H11|GLY|OADC agar plates with X-gal, KAN, and HYG. Blue, HYG and KAN resistant single colonies were inoculated in 7H9|GLY|OADC|Tween-80 broth containing KAN and then sub-cultured in media without antibiotic. A serial dilution of the sub-culture was performed (100 to 10^–3^) and 100 μl of each dilution was plated on 7H11|GLY|OADC agar plates with sucrose (2%), X-gal and HYG. White, sucrose tolerant single colonies were inoculated in 5 ml of supplemented 7H9 broth without any antibiotics and cultured for 7 days. The cultures were heat-killed, centrifuged, and the supernatant (DNA) was used for PCR amplification with in-house designed primer sets (Supplement Tables S2.1 and S2.2). Sanger sequencing of the PCR product was performed to confirm the presence of the *Rv0678* mutation of interest.

### Whole genome sequencing and bioinformatics

WGS of selected colonies was performed to confirm that no unintended mutations were acquired during the mutagenesis process. DNA was extracted from the selected mutant strains and the *Mtb* H37Rv wild type progenitor using a modified phenol–chloroform (CTAB; Hexadecyltrimethylammonium bromide) and sodium dodecyl sulphate (SDS) method^18,19^. Genomic DNA libraries were prepared using the Illumina Nextera Flex Library Preparation Kit according to manufacturer’s instructions (Illumina Inc., San Diego, CA, USA). WGS data was analysed using the in-house Universal Sequence Analysis Pipeline (USAP)^20^. Briefly, reads were mapped to *Mtb* H37Rv reference genome followed by trimming of adapters and low-quality bases with a Phred quality score of < 20. Trimmomatic^21^ was used to trim read length prior to alignment to *Mtb* H37Rv (GenBank NC000962.2). Three mapping algorithms (Burrows-Wheeler Aligner^22^, Novo Align^23^ and SMALT^24^) were used to ensure that real nucleotide differences were detected and false positive single nucleotide polymorphisms (SNP) are minimised for reads in repetitive regions. Following mapping, Genome Analysis Tool Kit (GATK)^25^ and SAMtools^26^ were used to identify SNPs, insertions, and deletions, compared to the *Mtb* H37Rv reference strain. Artemis^27^ was used for visual inspection of depth of mapping coverage of the aligned strains and to confirm the presence of the called mutations. SNP distance between mutant strains and the progenitor was determined using in-house Python scripts.

### Phenotypic drug susceptibility testing to determine the effect of mutagenesis

BDQ (Adooq Bioscience) was solubilised in Dimethyl Sulfoxide (DMSO) (Thermofisher Scientific) to prepare a stock concentration of 5.4 mg/ml. A volume of 500 μl of thawed glycerol stocks of bacterial cultures were added to MGIT culture tubes containing 7 ml of BBL media supplemented with 800 μl OADC. Tubes were incubated in the BACTEC MGIT 960 instrument at 37 °C until the culture flagged positive. MGIT cultures were incubated for a further 17 days at 37 °C, followed by blood agar purity testing and Ziehl–Neelsen (ZN) staining and microscopy. New MGIT tubes were inoculated with 500 μl of the extended MGIT culture and incubated in the BACTEC MGIT 960 machine at 37 °C until the 2nd day of MGIT culture positivity. A set of MGIT test tubes were prepared by supplementation with 800 μl of OADC and four different concentrations of BDQ (0.125, 0.25, 0.5, and 1.0 µg/ml). A growth control was prepared for each test strain by adding 100 μl from each of the day 2 positive MGIT cultures to 10 ml of sterile saline solution. A 500 μl aliquot of day 2 positive MGIT cultures was added into each of the drug-containing MGIT tubes and 500 μl of saline diluted culture for the different strains were added into different drug-free growth control MGIT tubes. We captured results of each experimental tube when 100 growth units (GU) was reached, or terminated the experiment when the growth control reached 400 GU. The *Mtb* H37Rv wild type strain was included as a susceptible control. A clinical isolate (BCCM/ITM, Antwerp, Belgium) with the p.Ala63Pro (c.187G>C) mutation in the *atpE* gene was included as BDQ resistant control. The BDQ phenotype was interpreted based on a critical concentration of 1.0 µg/ml^28^.

### In silico prediction of the phenotypic effect of mutagenesis

In addition to experimentally assessing the effect of the mutations of interest on the BDQ *Mtb* phenotype, we performed in silico analyses to predict the impact of mutations of interest on protein stability and interactions.

**Figure 1:**
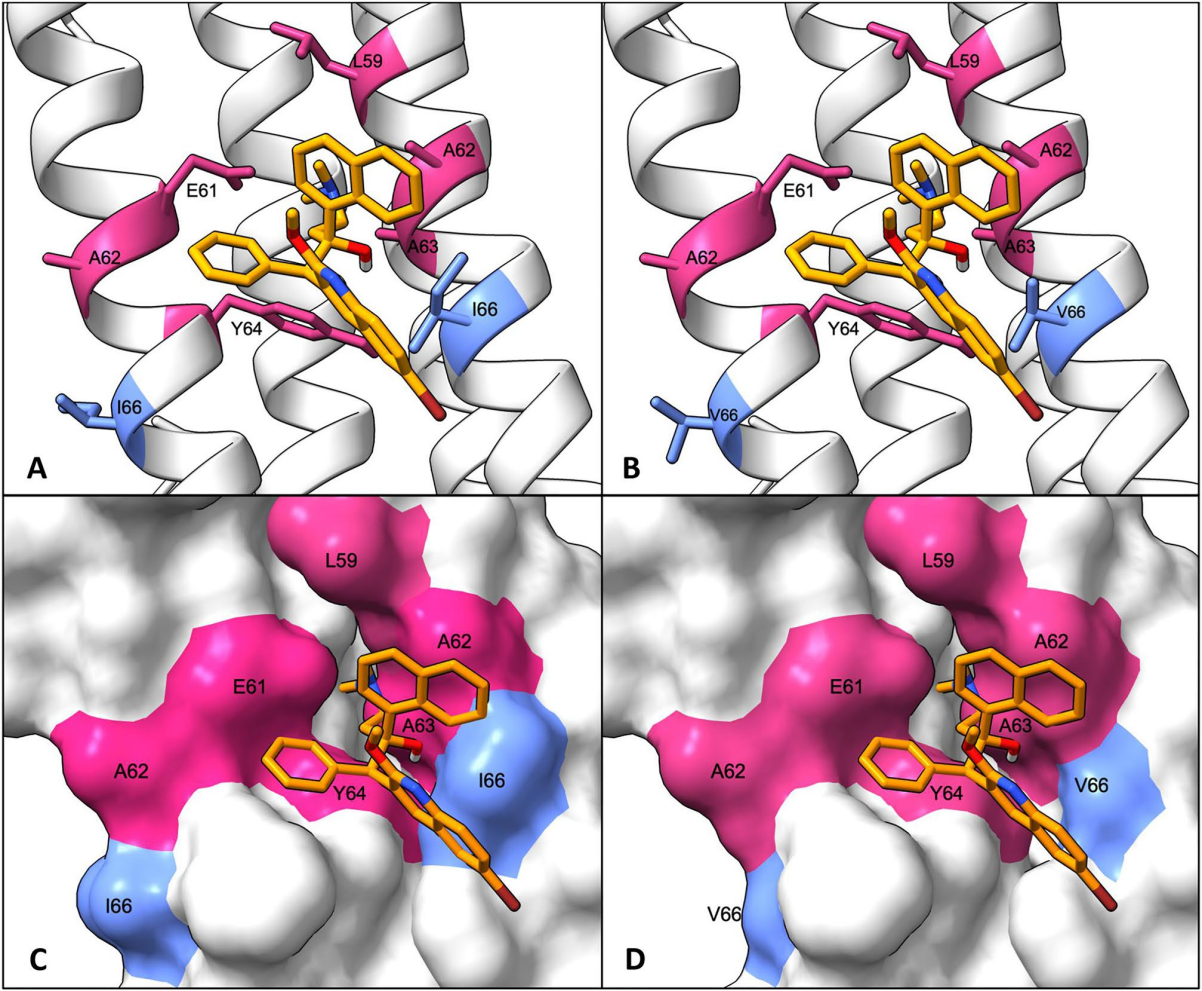
Structural modelling of the BDQ interaction with wild type and mutated *Mtb* ATP Synthase subunit C. Bedaquiline is shown in orange, the mutated residue is highlighted in blue, and the amino acids defining the binding cleft are highlighted in pink. (**A**) Cartoon representation of BDQ interacting with wild type ATP Synthase subunit C. (**B**) cartoon representation of BDQ interacting with mutated (Ile66Val) ATP Synthase subunit C. (**C**) Molecular surface representation of BDQ interacting with wild type ATP Synthase subunit C. (**D**) Molecular surface representation of BDQ interacting with mutated (Ile66Val) ATP Synthase subunit C.

The structure of *Mtb* ATP synthase subunit C was obtained through homology modelling with MODELLER^29^. A homology multi-chain (nonamer) model of *Mtb* ATP synthase subunit C was obtained by using the experimental structure of *Mycobacterium smegmatis* (*M. smeg.*) ATP synthase subunit C (protein databank [PDB] ID: 7JGC) as a guide structure, as that of *Mtb* was not available. Furthermore, the sequences of these two organisms share 84.9% sequence identity and 88.4% similarity for ATP synthase subunit C, with slight divergence limited to the termini. We also used the pairwise sequence alignment of the respective protein sequences (UniProt ID: P9WPS1 and A0R205)^30^ using the EMBOSS Needle^31^ tool. BDQ (DrugBank ID: DB08903) was docked onto the obtained structure using Autodock 4.2.6 through its graphical user interface MGLtools 1.5.7^32^ (Fig. 1).

**Figure 2:**
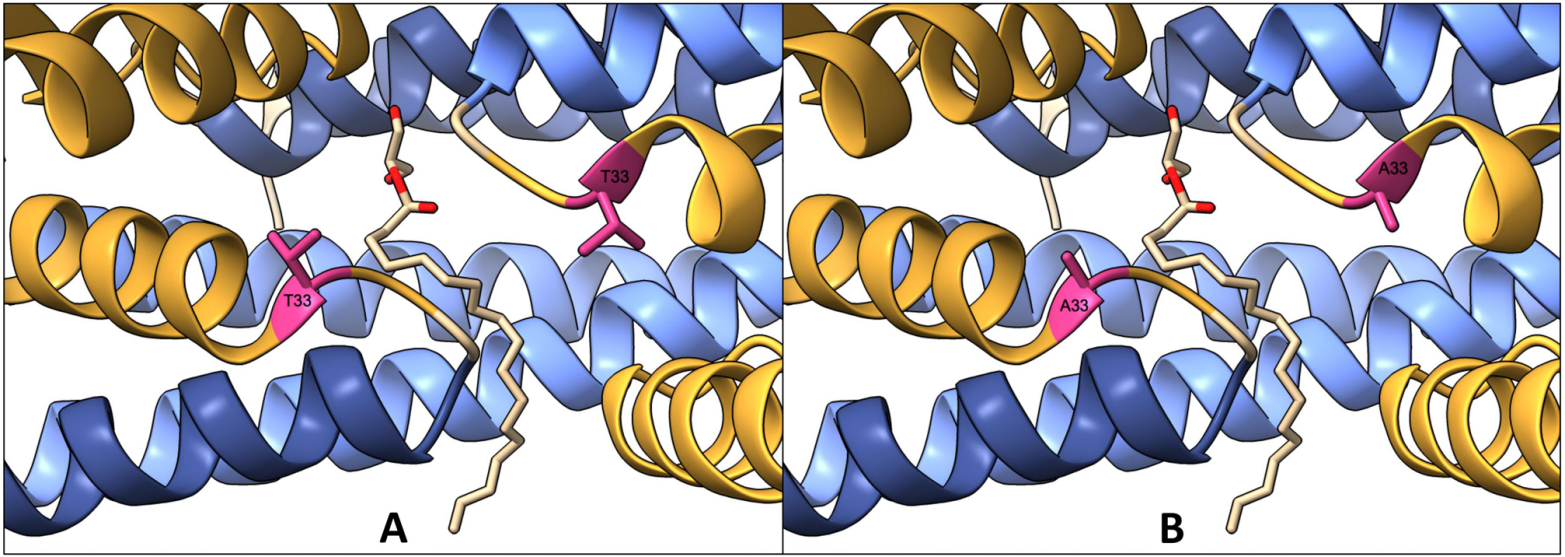
Structural modelling of MmpR dimer in complex with its fatty acid ligand, *1,3-dihydroxypropan-2-yl octadecenoate*. The dimerization domains are highlighted in blue, the DNA binding domains are highlighted in yellow, and the mutated residue is highlighted in pink. (**A**) Wild type MmpR dimer in complex with its fatty acid ligand. (**B**) Mutated (Thr33Ala) MmpR dimer in complex with its fatty acid ligand.

To assess the impact of the *Rv0678* Thr33Ala mutation on the protein structure and function of the transcriptional regulator encoded by *Rv0678*, we mapped the mutation on the previously determined *Mtb* MmpR experimental crystal structure (PDB ID: 4NB5)^33^ (Fig. 2).

All molecular structures were visualised in ChimeraX and protein structures were mutated using the ChimeraX rotamers tool^34^ (Figs. 1 and 2). The impact of the *atpE* Ile66Val and *Rv0678* Thr33Ala mutations on their respective protein structure and function was predicted using a suite of established mCSM tools (available at http://biosig.unimelb.edu.au/biosig). The change in protein stability was assessed using SDM2^35^, mCSM-Stability^36^, DUET^37^, and DynaMut2^38^. Changes in Protein–Protein affinity were evaluated using mCSM-PPI2^39^. Impact on the DNA binding affinity of the MmpR transcriptional regulator was predicted with mCSM-DNA^36^. A functional effect score was estimated using SNAP2, which integrates biophysical, evolutionary, and structural mutation features^40^, and an evolutionary conservation score was assigned using ConSurf^41^. Amino acid interchangeability for both mutations was assessed using the Grantham’s distance^42^. Finally, the SUSPECT-BDQ tool^43^ was used for BDQ phenotype prediction based on the log fold affinity change.

## Results

### Mutant generation

To experimentally assess the phenotypic effect of mutations in the two key BDQ candidate resistance genes on the BDQ MIC, site-directed mutagenesis was used to introduce the mutations into the *Mtb* H37Rv reference strain. The *Rv0678* c.97A>G (p.Thr33Ala) mutation was introduced by homologous recombination and the *atpE* c.196A>G (p.Ile66Val) mutation through homologous recombineering. In the recombineering experiment, 33 colonies were screened with PCR amplification and Sanger sequencing to confirm the presence of the *atpE* p.Ile66Val mutation. Of the 33 colonies, only two contained the *atpE* mutation; one colony (C11) had a mixed population of wild type (CAT) and mutant (CGT) alleles and one colony (C14) had a pure *atpE* p.Ile66Val mutant population (Supplement Fig. S3.1A).

For the homologous recombination procedure, of the 62 colonies screened with PCR amplification and Sanger sequencing: five had a pure *Rv0678* p.Thr33Ala mutant population and twelve had mixed populations of wild type and mutant alleles (Supplement Fig. S3.1B). Colonies C15 and C46 were selected for further analyses.

### Whole genome sequencing

The H37Rv progenitor strain, colonies C14, containing the *atpE* mutation, C15 and C46, containing the *Rv0678* mutation, were assessed by whole genome sequencing (WGS) to identify additional mutations that may have been acquired during the mutagenesis procedure. While all selected strains had acquired at least one additional mutation during the site-directed mutagenesis experiments, none had acquired a mutation in any of the BDQ candidate resistance genes, other than the introduced mutations (Table 1). The *atpE* mutant strain (C14) acquired one additional synonymous mutation (*Rv2235* c.207G>A; p.Ser69Ser) in 100% of the reads. Both *Rv0678* mutated strains (C15 and C46) acquired the same four additional mutations, including one intergenic (*Rv0439c* c.-39C>A), one synonymous (*Rv1662* c.4215C>T; p.Ala405Ala), and two missense mutations (*Rv0679c* c.426C>G; p.Asn142Lys and *Rv2933* c.2531T>C; p.Leu844Pro). All of these unintended mutations were fixed, and present in all of the sequenced isolates, suggesting that the mutations arose early on in the mutagenesis process. The H37Rv progenitor strain acquired one mutation (c.979A>G; p.Thr327Ala) in the *Rv3507* gene in 67% of reads, however, the region is highly variable and this mutation may represent sequencing- or mapping error.

**Table 1:** Whole genome sequencing: introduced mutations.

Colony	DNA mutation	Protein mutation	Gene	Gene function
C14 (*atpE* mutant)	196A>G^#^	Ile66Val^#^	*atpE* (*Rv1305*)	ATP synthase subunit C
	207G>A*	Ser69Ser	*Rv2235*	Function unknown; survival in macrophages, probable conserved transmembrane protein
C15 and C46 (*Rv0678* mutant)	− 39C>A	NA	*Rv0439c*	Function unknown; probable dehydrogenase/reductase
	97A>G^#^	Thr33Ala^#^	*Rv0678*	MmpR; transcriptional regulator of *mmpS5/mmpL5*
	426C>G	Asn142Lys	*Rv0679c*	Function unknown; conserved threonine rich protein
	4215C>T*	Ala1405Ala	*Rv1662*	Pks8; potentially involved in synthesis of a polyketide molecule
	2531T>C	Leu844Pro	*Rv2933*	PpsC; phenolpthiocerol and phthiocerol dimycocerosate (dim) biosynthesis
H37Rv	979A>G	Thr327Ala	*Rv3507*	PE_PGRS53; function unknown

^#^The desired mutations introduced by mutagenesis.

^*^Synonymous mutation.

### Phenotypic testing

To determine the effect of the introduced mutations on the BDQ phenotype, MIC values were determined in triplicate for colonies C14 (*atpE* mutation), C15 and C46 (*Rv0678* mutation), H37Rv as the susceptible control, and a clinical BDQ resistant isolate containing the *atpE* Ala63Pro mutation (*Rv0678* wild type) (Table 2). The *atpE* mutant strain C14 had a susceptible BDQ MIC of 0.25–0.5 µg/ml, comparable to the MIC of the H37Rv reference strain (0.25–0.5 µg/ml). The *Rv0678* mutant strains C15 and C46 and the resistant control were phenotypically resistant to BDQ (MIC > 1.0 µg/ml), having reached 100 GU before the growth control had reached 400 GU (Supplement Fig. S3.2).

**Table 2:** MIC reading after 7 days of incubation.

Strain	Mutation	Repeat	BDQ concentration (µg/ml)				MIC	BDQ phenotype*
			0.125	0.25	0.5	1.0		
C14	*atpE* Ile66Val	1	R	R	S	S	0.5	Susceptible
		2	R	R	S	S	0.5	Susceptible
		3	R	S	S	S	0.25	Susceptible
C15	*Rv0678* Thr33Ala	1	R	R	R	R	> 1.0	Resistant
		2	R	R	R	R	> 1.0	Resistant
		3	R	R	R	R	> 1.0	Resistant
C46	*Rv0678* Thr33Ala	1	R	R	R	R	> 1.0	Resistant
		2	R	R	R	R	> 1.0	Resistant
		3	R	R	R	R	> 1.0	Resistant
H37Rv	Wild type	1	R	S	S	S	0.25	Susceptible
		2	R	R	S	S	0.5	Susceptible
		3	R	S	S	S	0.25	Susceptible
Resistant control	*atpE* Ala63Pro	1	R	R	R	R	> 1.0	Resistant
		2	R	R	R	R	> 1.0	Resistant
		3	R	R	R	R	> 1.0	Resistant

*BDQ* bedaquiline, *MIC* minimal inhibitory concentration, *R* resistant, *S* susceptible.

*Based on 1.0 µg/ml cut-off.

We noted a marked decrease in the growth of the *Rv0678* mutant, compared to the wild-type, with the no-drug control of the mutant only reaching 400 GU after 13 days, compared to the WT at approximately 7 days (Fig. S3.2). This may indicate a fitness cost conferred by the *Rv0678* Thr33Ala mutation. The *atpE* mutant had no appreciable growth deficit.

### In silico analysis

To assess the effect of the introduction of the *Rv0678* p.Thr33Ala and *atpE* p.Ile66Val mutation in silico, structural analyses were performed. BDQ interacts with two ATP synthase subunit C protomers that form a binding cleft defined by amino acids Glu61 and Tyr64 of one protomer and Leu59, Ala62, Ala63 and Ile66 of the other protomer (Fig. 1). The best scoring docking conformation (root-mean-square deviation = 333.110 Å, estimated free energy of binding = − 7.03 kcal/mol) of BDQ on the homology multi-chain (nonamer) model of *Mtb* ATP synthase subunit C was consistent with the BDQ–ATP synthase interaction of the *Mycobacterium smegmatis* experimental structure. The Ile66 residue of interest is located on the C-terminal end of the binding cleft with a measured distance of 3.217 Å to the docked ligand (BDQ). The impact of the Ile66Val mutation on *Mtb* ATP synthase subunit C stability was consistent across the four mCSM prediction tools, which all predicted reduced stability or a destabilizing effect (Table 3). While the mCSM-PPI2 tool predicted increased protein–protein affinity (ΔΔG 0.011), the SUSPECT-BDQ predicted the phenotypic outcome of the Ile66Val mutation as resistant, given an affinity fold change (ΔΔG) of − 0.469 log. The predicted evolutionary conservation score calculated with ConSurf was − 1.220, indicating that the affected residue is highly conserved among homologous sequences. The predicted SNAP2 score of 22 indicated that the p.Ile66Val mutation would have an intermediate impact on ATP synthase function, consistent with other prediction methods. The low Grantham’s distance indicates that isoleucine and valine are physiochemically similar amino acids that are interchangeable with little impact on the protein structure.

**Table 3:** Mutation impact on protein stability and function.

Tool	Predicted feature	*atpE* p.Ile66Val		*Rv0678* p.Thr33Ala	
		Result	Interpretation	Result	Interpretation
SDM2	Change in protein stability (ΔΔG)	− 0.35	Reduced stability	− 0.08	Reduced stability
mCSM-Stability	Change in protein stability (ΔΔG)	− 0.87	Destabilizing	− 1.07	Destabilizing
DUET	Change in protein stability (ΔΔG)	− 0.59	Destabilizing	− 0.92	Destabilizing
DynaMut2	Change in protein stability (ΔΔG)	− 1.24	Destabilizing	− 0.74	Destabilizing
mCSM-PPI2	Protein–Protein affinity change (ΔΔG)	0.011	Increasing affinity	− 0.30	Decreasing affinity
SNAP2	Functional effect (score)	22	Intermediate	− 33	Neutral
ConSurf	Evolutionary conservation (score)	− 1.22	Conserved	− 0.10	Average
SUSPECT-BDQ	BDQ phenotype prediction (log fold affinity change)	− 0.46	Resistant	*NA*	*NA*
mCSM-DNA	Protein-DNA affinity change (ΔΔG)	*NA*	*NA*	− 1.35	Destabilizing
Grantham’s distance	Amino acid interchangeability	29	Low, similar amino acids	58	Intermediate

*BDQ* bedaquiline.

Mapping of the *Rv0678* p.Thr33Ala mutation on the *Mtb* MmpR protein crystal structure revealed that the mutation is located in the N-terminal DNA binding domain (Fig. 2). The impact of the Thr33Ala mutation on *Mtb* MmpR stability was consistent across the four mCSM prediction tools, which all predicted reduced stability or a destabilizing effect (Table 3). The predicted evolutionary conservation (ConSurf) score of -0.104 indicated that the affected residue is neither variable nor conserved. The mCSM-PPI2 tool predicted a reduction in protein–protein affinity (ΔΔG = − 0.30 kcal/mol) and the mCSM-DNA tool indicated that the Thr33Ala mutation would have a substantial impact on the DNA binding affinity of the MmpR transcriptional repressor (ΔΔG = − 1.345 kcal/mol). Interestingly, the predicted SNAP2 score of − 33 indicated that the Thr33Ala would have no effect on MmpR function. The intermediate Grantham’s score indicates that threonine and alanine are physicochemically dissimilar amino acids, meaning that interchanging these amino acids could have a substantial impact on the protein structure.

## Discussion

The large number of unique mutations observed in the BDQ candidate resistance genes poses a challenge for accurate molecular detection of BDQ resistance, as very few mutations have been statistically associated with BDQ resistance or susceptibility^8^. Furthermore, seemingly conflicting data hamper the prediction of the clinical impact of novel or rare mutations. For example, the *atpE* p.Ile66Val mutation has been reported in a clinical isolate in Australia^11^ as having increased but still susceptible BDQ MIC of 0.125 µg/ml on REMA. This is unusually low for *atpE* mutations as these typically confer high level resistance, which is not surprising given the essential function of ATP synthase in the electron transport chain^44^. In the same vein, the *Rv0678* p.Thr33Ala mutation has been observed in in vitro isolates with very high MIC values (8 µg/ml on MGIT and > 2 µg/ml on 7H10 medium), both in combination with other mutations and as a single mutation^10,12^, while many *Rv0678* mutations in clinical BDQ resistant isolates tend to result in a low level of BDQ resistance^8^. Both mutations (*Rv0678* p.Thr33Ala and *atpE* p.Ile66Val) are important as they have been reported in clinical isolates^3,11^.

To improve our understanding of the molecular mechanisms of BDQ resistance, we introduced two mutations (*atpE* p.Ile66Val and *Rv0678* p.Thr33Ala) in the H37Rv reference strain to experimentally assess their functional impact. Phenotypic analysis of the resulting mutants supports the clinical observation that the *atpE* Ile66Val mutation does not confer resistance as the mutant strain had a BDQ MIC comparable to that of the H37Rv reference strain^11^, while the *Rv0678* p.Thr33Ala appears to indeed confer BDQ resistance.

The structural modelling and feature prediction analyses shed light on why this seemingly counterintuitive finding of BDQ susceptibility of an *atpE* mutant is correct. The *atpE* p.Ile66Val mutation causes a slight destabilizing effect on the ATP synthase subunit C protein stability. However, even though the *atpE* p.Ile66Val mutation is located on the edge of the BDQ binding site, the substituting isoleucine for valine is likely not disruptive enough, as evidenced by the low Grantham’s distance, to have a substantial impact on the interaction with BDQ, allowing the strain to maintain its susceptible phenotype^42,45^. It is important to note that the SUSPECT-BDQ tool erroneously classified the *atpE* p.Ile66Val mutation as resistant, likely based on the short distance of the affected residue to the bound ligand (BDQ)^43^. This highlights the importance of thorough validation of deep learning classification models.

It has recently been suggested that there are three distinct mechanisms through which mutations in the *Rv0678* gene might affect the BDQ phenotype, including protein stability, protomer interaction, and DNA interaction^46^. This highlights the importance of a comprehensive in silico assessment of the effect of the introduction of a mutation. Our feature prediction further shows that the *Rv0678* p.Thr33Ala mutation had a small destabilizing effect on the MmpR protein structure, to a similar extent as the *atpE* mutation. However, a large decrease in DNA-binding affinity was predicted, consistent with the location of the mutation in the N-terminal DNA binding domain. This decrease in binding affinity is likely the cause of the change to a BDQ resistant phenotype.

Taken together, our results support clinical observations that the *Rv0678* p.Thr33Ala mutation causes a decrease in BDQ susceptibility, while the *atpE* Ile66Val mutation does not (Table 4).

**Table 4:** Summary of results.

	*atpE* Ile66Val	*Rv0678* Thr33Ala
Clinical observations	Not BDQ resistant^a^	BDQ resistant^b^
Mutant	Not BDQ resistant	BDQ resistant
In silico analysis	Minimal disruption of BDQ-ATP synthase interaction	Substantial disruption of DNA binding affinity of Rv0678

^a^Martinez et al. Mutations associated with in vitro resistance to bedaquiline in Mycobacterium tuberculosis isolates in Australia. Tuberculosis (Edinb), 2018.

^b^Andries et al. Acquired Resistance of Mycobacterium tuberculosis to Bedaquiline. PLoS One, 2014.

Although secondary unwanted mutations were acquired in the host genome during the mutagenesis procedure, the chance of acquiring these mutations remains lower compared to other in vitro methods, where drug resistance mutations are elicited through exposure to low concentrations of antibiotics in culture and subsequent selection using lethal drug concentrations^12,46^. These methods also require additional experiments to rule out the possibility of secondary mutations being culture-selected due to drug pressure. Furthermore, in vitro exposure methods result in variable profiles of resistance mutations, often favoring mechanisms that are not clinically relevant, while homologous recombineering and recombination allow for introduction of specific predetermined mutations in the host genome^47^.

To our knowledge, our study is the first to introduce mutations in BDQ candidate resistance genes in *Mtb* to assess the phenotypic effect of potential resistance markers and to investigate the structural changes in silico to investigate the causal changes underlying the resulting phenotype. Despite the strengths of our study, several limitations should be noted. First, we selected only two mutations in two BDQ resistance conferring genes even though many mutations in five different genes are believed to play a role in BDQ resistance. Future experiments should be conducted to expand the repertoire of mutations investigated, including similar mutations with apparently opposite effects, such as the *atpE* p.Ile66Met mutation reported by Le Ray et al. which apparently resulted in an elevated MIC in a clinical isolate^48^. Given the structural difference between Isoleucine and Valine, compared to Methionine, with the latter containing a Sulphur molecule, while lacking the branched structure of the former, it is conceivable that the effect of Methionine at this position is very different. Second, our study did not include experimental validation of the mechanism of resistance conferred by the *Rv0678* p.Thr33Ala mutation. One approach would be to restore the mutated strains back to wild type using the same method, however this carries the risk of adding further unintended mutations that may complicate interpretation of results. Additionally, selection of the wild type would be problematic due to the limited availability of selectable markers for use in *Mtb*. BDQ efflux experiments may be carried out for the *Rv0678* mutant given that other studies were able to show that *Rv0678* mutations upregulates the MmpL5/S5 efflux pump, which exports BDQ, but none of these experiments included the *Rv0678* p.Thr33Ala mutation^3,49,50^. Taken together, the findings of these studies and our in silico analysis indicating the impact on the DNA binding affinity of the MmpR transcriptional repressor, upregulated BDQ efflux seems a plausible explanation for resistance conferred by the *Rv0678* p.Thr33Ala mutation. Third, other mutations, most likely hitchhiking mutations^46^, were acquired during the experiments in all strains, including the H37Rv reference strain, confirming that *Mtb* can evolve in a controlled in vitro environment^13^. Specifically, the *atpE* p.Ile66Val mutant strain acquired a synonymous mutation in the *Rv2235* gene, which encodes an uncharacterized SURF1-like protein with an unknown function and has not been described in context of BDQ resistance or ATP synthase. The *Rv0678* Thr33Ala mutant strains acquired four mutations, of which one (c.426C>G; p.Asn142Lys) was located in the *Rv0679c* gene which encodes an uncharacterized threonine-rich protein with an unknown function and has also not been described in relation to BDQ resistance. According to literature, this mutation has been observed in clinical isolates of the Beijing genotype and appears to influence the immune response^51,52^. Given that this mutation has been classified as a lineage marker, it is unlikely to affect the BDQ phenotype^53^. None of the other three acquired mutations, *Rv0439c* c.-39C>A, *Rv1662* c.4215T>C, and *Rv2933* c.2531T>C, have been described before. The c.979A>G mutation in the *Rv3507* gene, which forms part of the proline-glutamine-polymorphic GC-rich sequences (PE/PE-PGRS) gene family, observed in the H37Rv reference genome^13^ likely indicates genetic homogeneity of the reference genome. These findings highlight the importance of performing WGS analyses to investigate all mutations introduced during site-directed mutagenesis experiments. Furthermore, while unlikely, the possibility that these secondary mutations influence the BDQ phenotype cannot be excluded. Definite proof that the *Rv0678* p.Thr33Ala mutation causes a decrease in BDQ susceptibility would require complementation experiments to demonstrate full restoration of the phenotype.

Both homologous recombination and homologous recombineering were effective to achieve the desired mutation. The two techniques have different strengths and limitations. In general, the main advantage of homologous recombineering over recombination is the simplicity of design (a single-stranded oligonucleotide carrying the mutation of interest) and execution (single selection step), as well as low cost. In contrast, despite complicated and time-consuming experimental procedures, homologous recombination offers more precise selection, and a high frequency of recombination with lower levels of illegitimate recombination. Our data suggests that homologous recombination may be slightly more efficient to produce pure mutant colonies (62 colonies produced of which five were pure mutants vs. 33 colonies produced, of which one was a pure mutant). However, the two-step recombination process appears to introduce more unintended mutations (four vs one).

## Conclusion

We have shown that homologous recombination and recombineering can be successfully used to assess the phenotypic impact of genomic mutations on BDQ MIC in vitro. In combination with functional validation of the introduced mutations, these methods can be powerful tools to increase our understanding of specific BDQ resistance causing mutations, which is vital to providing appropriate treatment to patients with rifampicin resistantTB.

## Supplementary Information

Supplementary Information.

## Data Availability

Sequencing reads have been deposited at the European Nucleotide Archive (project Accession Number: PRJEB52049; https://www.ebi.ac.uk/ena/browser/view/PRJEB52049). Protein structures used for in silico predictions are available upon request.
