# Supplementary material for: Site-directed mutagenesis of Mycobacterium tuberculosis and functional validation to investigate potential bedaquiline resistance-causing mutations
Source: Sci Rep. 2023 Jun 6;13:9212. doi: 10.1038/s41598-023-35563-0 (PMC10244393; doi:10.1038/s41598-023-35563-0)
Supplement: Supplementary file 1 — Supplementary Information. [file 41598_2023_35563_MOESM1_ESM.docx]

1. **Homologous Recombineering**

# Table S1.1: *Mycobacterium tuberculosis* homologous recombineering ssDNA synthetic oligonucleotides

| **ssDNA oligonucleotide** | **Sequence (5-3)** | **size** | **Target** | **Purpose** |
| --- | --- | --- | --- | --- |
| RcbMtb_atpEI66V_lag | AAGACGAACAGCGCCATAAACGCCAGGTTG{ACG}AAGTATGCCGCCTCAACCAAACCGACGGTG | 63bp | *atpE* | c.196A>G |
| JCV 198 | CCCTGTTACTTCTCGACCGTATTGATTCGGATGATTCCTACGCGAGCCTGCGGAACGACCAGG | 63bp | *php* cassette on pJV75amber | Restore hygR cassette |
| Mtb_atpE_B_Fwrd_SEQprim | GAGCCATCAAGGAGGATAAGG | 299bp | *atpE* | PCR amplification of *atpE* gene |
| Mtb_atpE_B_Rev_SEQprim | TGCTACCTATTGCAACCATTTG | 299bp | *atpE* | PCR amplification of *atpE* gene |
| PJV75_KANR_F | ATTCAACGGGAAACGTCTTG | 700bp | *aph* | PCR amplification of pJV75amber (confirm presence of plasmid) |
| PJV75_KANR_R | CGAGGCAGTTCCATAGGATG | 700bp | *aph* | PCR amplification of pJV75amber (confirm presence of plasmid) |

# Table S1.2: Thermocycling conditions for amplification of the *atpE* gene using Qiagen Master mix HotStart Taq polymerase

| Thermocycling step | Temperature (°C) | Time | Number of cycles |
| --- | --- | --- | --- |
| 1) Taq activation | 95 | 5 mins | 1 |
| 2) Denaturation | 94 | 1 min | 35 - 40 |
| 3) Annealing | 55°C | 1 – 2 mins |  |
| 4) Elongation | 72 | 1 min |  |
| 5) Extension | 72 | 10 mins | 1 |
| 6) Holding step | 4 | - | - |

**Figure S1:** **General workflow of homologous recombineering.** Homologous recombineering involves transformation of Mtb cells with pJV75amber carrying selectable markers and an inducible recombinase protein gp61. Following selection of transformed cells, the recombinase is induced with acetamide. The cells are then transformed by electroporation with two ssDNA oligonucleotides: one homologous to the genomic region of interest, carrying the nucleotide change centrally; the other (JVC198) carrying a hygromycin resistance cassette for confirmation of recombineering event on the plasmid vector. White hygR colonies are screened for the mutation of interest.

1. **Homologous Recombination**

The flexible cassette in the two-step plasmid cloning system for homologous recombination was developed by Parish and Stoker (Parish and Stoker, 2000). This technique uses the traditional cloning method of restriction enzyme digestion and ligation to insert homologous DNA fragment into the p2NIL suicide plasmid vector’s multiple cloning site (MCS). The selectable and counter-selectable markers are then added using a unique PacI restriction enzyme site in p2NIL (Kendall and Frita, 2009).

### Design of primers to amplify homologous DNA regions.

An online bioinformatics tool called Primer3plus (Untergasser et al., 2007), was used to design PCR primers. All the PCR primers (Table S2.1) were commercially synthesized by Integrated DNA Technologies (WhiteHead Scientific) at 100-nm scale with PAGE purification for 41bp sequences (primers 3 and 4; Table S2.1) and at 25-nm scale with standard desalting for 20bp to 32bp sequences (primers 3 and 4; Table S2.1)Homologous DNA insert carrying the desired mutation was generated by overlap PCR primers using two pairs of primers (primers 1 to 4; Table S2.1) in a three-step PCR amplification process. The outer primers (primer 3 and 4, Table S2.1) bind at the 5’ and 3’ ends of the upstream and downstream regions of the gene and insert restriction enzyme sites. The outer primers consisted of 20bp sequence homology to the gene region, 6bp restriction enzyme sites, and an extra 6bp non-coding sequence on the outer ends of the primers. This creates additional sequence allowance for direct restriction enzyme excision of the amplified DNA fragment during cloning experiments. The central primers (primers 1 and 2, Table S2.1) are complementary and carry the single nucleotide change Thr > 33> Ala of interest. They overlap in the middle of the upstream and downstream regions, such that the nucleotide base change is incorporated into the center of the dsDNA fragment.

### **Generation of** *rv0678* homologous region containing the Thr33Ala mutation.

Generation of the dsDNA fragment involved three steps. Firstly, The PCR amplification of the upstream and downstream regions, separately. Secondly, PCR annealing of two overlapping DNA fragments and extension of *rv0678* fragment, and thirdly PCR amplification of the annealed *rv0678* fragment and full extension of the dsDNA strand carrying the gene. Genomic DNA from *M. tuberculosis* H37Rv was used as a DNA template in a 25μl PCR reaction mix using Phusion high fidelity Hotstart *flex* polymerase (Bio-lab) for PCR amplification of the DNA carrying the *rv0678* gene of interest (Table S2.3). Thermocycling conditions were the same for all PCR reactions (Table S2.2). The first amplification step (Table S2.3) was carried out with two pairs of primers to amplify two DNA fragments: the upstream region with primers 2 and 3 (Table S2.1) and downstream region with primers 1 & 4 (Table S2.1).

In the second step, annealing and extension of the upstream and downstream regions of *rv0678* fragment was performed using cleaned up and purified PCR product from the amplified upstream and downstream DNA fragment (Table S2.4).

In the last amplification step (Table S2.5), overlapped *rv0678* fragment purified PCR product was mixed with primers 3 & 4 (Table S2.1), to amplify and extend the full-length of the dsDNA strand carrying the *rv0678* Thr33Ala fragment.

# Table S2.1: Mycobacterium tuberculosis primer sets for homologous recombination to introduce the Rv0678 c.97A>G (p.Thr33Ala) mutation

| **Primer nr** | **PCR Primer** | **Sequence (5-3)** | **Product size** | **Target** | **Purpose** |
| --- | --- | --- | --- | --- | --- |
| 1 | Mtb_Rv0678_T33A_For_ampyP | ATTTCGAGTCCAGGAGTTTGGCTCGGTTGGCGGGTCGATT{G} | 1413bp | *Rv0678* | Insert a97g (overlap PCR) |
| 2 | Mtb_Rv0678_T33A_Rev_ampyP | CAATCGACCCGCCAACCGAGCCAAACTCCTGGACTCGAAA{T} | 1413bp | *Rv0678* | Insert a97g (overlap PCR) |
| 3 | RcmbFrag_Rv0678_Fwrd_digPrim | ACAGCG{*AAGCTT*}CAGGTTGGTCGCG AAGGTCG | 2825bp | HindIII site | Insert restriction site into plasmid by PCR |
| 4 | RcmbFrag_Rv0678_Rev_digPrim | CGTACA{*GGTACC*}ACGTACGCTCAATCAAGGCC | 2825bp | kpnl site | Insert restriction site into plasmid by PCR |
| 5 | Mtb_Rv0678_Fwrd_SEQ | GCGACCACAACCAGGATGA | 885bp | *Rv0678* | PCR amplification of *Rv0678* for Sanger sequencing |
| 6 | Mtb_Rv0678_Rev_SEQ | CTCGGACAACACGCTGACC | 885bp | *Rv0678* | PCR amplification of *Rv0678* for Sanger sequencing |

# Table S2.2 Thermocycling conditions for PCR amplification, overlap and extension of *rv0678* dsDNA fragment with HindIII and KpnI restriction site

| Thermocycling steps | Temperature | Time | No of cycles |
| --- | --- | --- | --- |
| Step 1: Enzyme (Taq) activation | 98°C | 30 seconds | 1 |
| step2: Denaturation | 98°C | 10 seconds |  |
| step 3: Annealing | 69°C | 2 mins | 35 - 40 |
| step 3: Elongation | 72°C | 30 seconds |  |
| step 4: Extension | 72°C | 10 mins | 1 |
| Step 4: Holding step | 4°C | infinite | 1 |

# Table S2.3 Phusion high fidelity Hotstart flex polymerase 25μl PCR reaction used to amplify upstream and downstream regions to incorporate the restriction enzyme sites (step 1)

| Reagents | Final Concentration | 25μl Reaction (1x) |
| --- | --- | --- |
| Nuclease free water | Add to the final volume | 13.50μl |
| 5x Phusion HF buffer | 1x | 5.0μl |
| 10μM dNTPs mix | 200uM of each 2.5mM | 4.0μl |
| Forward primer (10μM) * | 0.5μM | 1.25μl |
| Reverse primer (10μM) ** | 0.5μM | 1.25μl |
| DMSO | 3% | 0.75μl |
| Phusion Hot start Flex Taq (10 units/μl) | 1unit/50µlrxn | 0.25μl |
| Subtotal volume |  | 24μl |
| DNA | 100ng | 1μl |
| Total reaction volume |  | 25μl |

* RcmbFrag_*Rv0678*_Fwrd_digPrim for upstream reaction or Mtb_*Rv0678*_T33A_For_ampyP for downstream reaction.

** Mtb_*Rv0678*_T33A_Rev_ampyP for upstream reaction or RcmbFrag_*Rv0678*_Rev_digPrim for downstream reaction.

# Table S2.4 Phusion high fidelity Hotstart flex polymerase 50μl PCR reaction mix for fragment annealing (step 2)

| Reagent | Final Concentration | 50 μl Reaction (1x) |
| --- | --- | --- |
| Nuclease free water | Add up to final volume | make up final volume |
| 5x Phusion HF buffer | 1x | 10μl |
| 10uM dNTPs mix | 200uM of each 2.5mM | 4μl |
| DMSO | 3% | 1.5μl |
| Phusion Hot start Flex Taq polymerase (10 units/ul) | 1unit/50μl reaction | 0.5μl |
| Subtotal volume |  | 46μl |
| Amplified Upstream DNA fragment: cleaned up and purified PCR product | 100ng | 2μl |
| Amplified Downstream DNA fragment: cleaned up and purified PCR product | 100ng | 2μl |
| Total reaction volume |  | 50μl |

# Table S2.5 Phusion high fidelity Hotstart flex polymerase 50μl PCR reaction for fragment extension (step 3)

| Reagent | Final Concentration | 50μl Reaction (1x) |
| --- | --- | --- |
| Nuclease free water | Add up to final volume | 2.5μl |
| 5x Phusion HF buffer | 1x | 2.5μl |
| RcmbFrag_Rv0678_Fwrd_digPrim:  5’ - ACAGCG{AAGCTT}CAGGTTGGTCGCGAAGGTCG - 3’ | 0.5uM | 2.5μl |
| RcmbFrag_Rv0678_Rev_digPrim:  3’ - CGTACA{GGTACC}ACGTACGCTCAATCAAGGCC - 5’ | 0.5μM | 2.5μl |
| Subtotal volume |  | 10μl |
| Amplified overlapped DNA fragment: purified PCR product | 100ng | 40μl |
| Total reaction volume |  | 50μl |

# Table S2.6: Plasmid vectors for homologous recombineering

| **Plasmid** | **Characteristics** | **Source** |
| --- | --- | --- |
| pJET1.2 | Subcloning sequencing vector used for selection (ampR) | Addgene |
| p2NIL | Expression cloning vector for selection of construct (kanR) | Addgene |
| pGOAL19 | Lambda RED recombinase cloning vector used for expression of hygR *LacZ* and *SacB* | Addgene |
| pJET1.2_*rv0678* | Subcloning sequencing vector to confirm *rv0678* variant (ampR) | This study |
| p2NIL_r*v0678* | Vector used for the expression of *rv0678* (kanR) | This study |
| p2NIL_*rv0678_*pGOAL19 | Final vector used to deliver *rv0678* dsDNA fragment into host chromosome (kanR*,* hygR, X-GAL) | This study |

**Figure S2.** **A** **General workflow of homologous recombination.** Wild type *Rv0678* DNA fragment was amplified from H37Rv through three PCR amplification steps. First, the amplification of the up- and downstream regions of the *Rv0678* DNA fragment to insert restriction enzyme sites at both 5’ and 3’ ends. Second, the overlapping of the two DNA fragments to incorporate a single nucleotide base substitution Thr33Ala in the center of the two overlapped DNA fragments. Third, the full extension of the overlapped and joined DNA fragments flanking the mutated *Rv0678* Thr33Ala gene of interest. The mutated *Rv0678* gene fragment generated by PCR was first expressed in pJET1.2 and then excised through restriction enzyme digest. The excised mutated *Rv0678* gene fragment was inserted into p2NIL (carrying a kanamycin resistance gene), which is then combined with pGOAL19 to create the p2NIL_rv0678_pGOAL19 suicide vector. Mtb cells are transformed with the suicide plasmid DNA, to facilitate single-crossover (SCO) integration of the plasmid, stimulated in the presence of kanR, hygR or X-galactosidase (LaZ). The integrated suicide plasmid is removed in a double crossover counter selection with sucrose (SacB) for white colonies in the blue colonies background. The white colonies are screened for the mutation of interest. **B Vector map of p2NIL pGOAL19** used for homologous recombination.


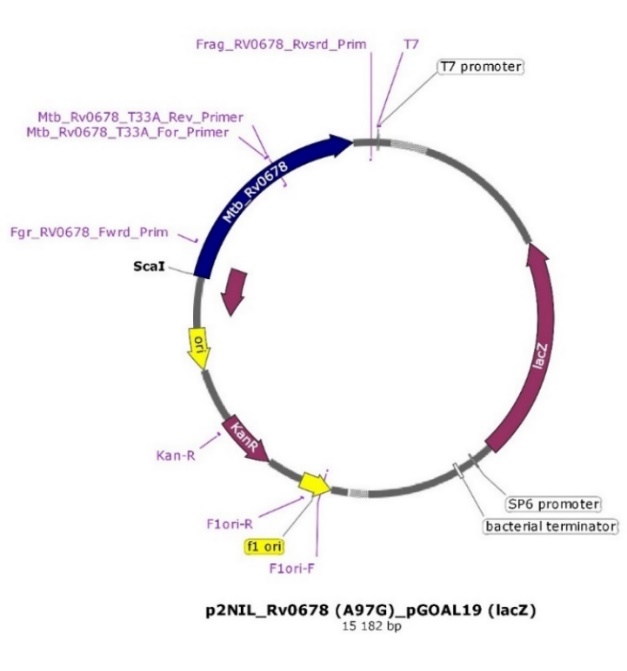


**A**

**B**

1. **Results**

**A**


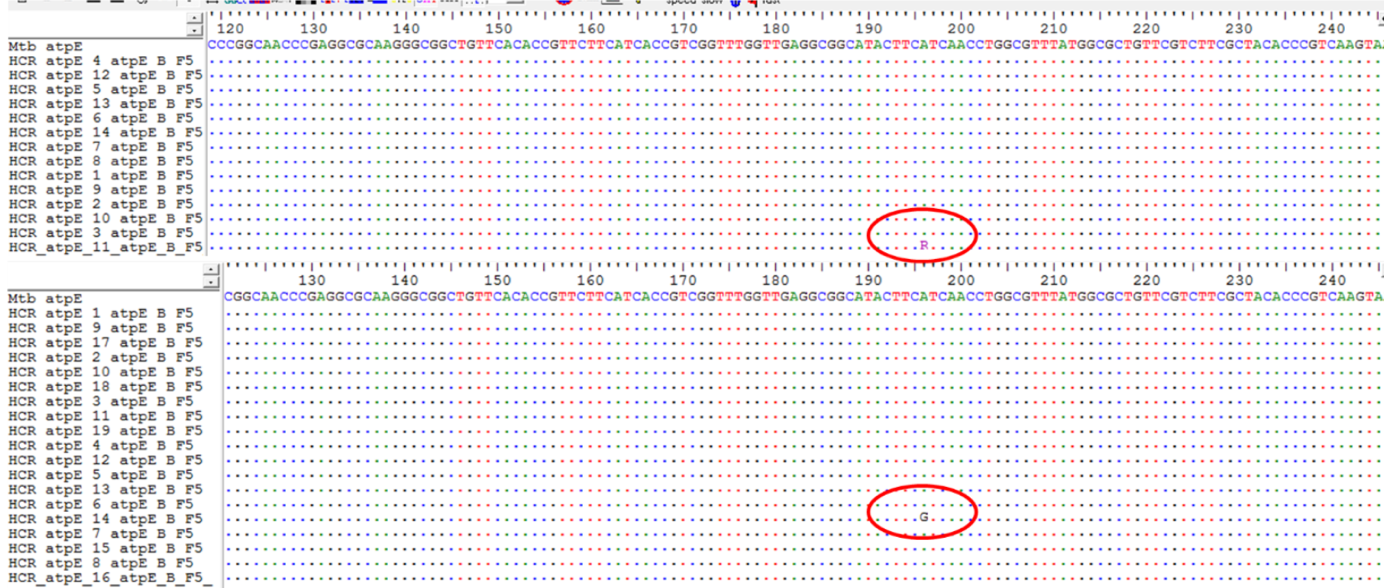


**B**


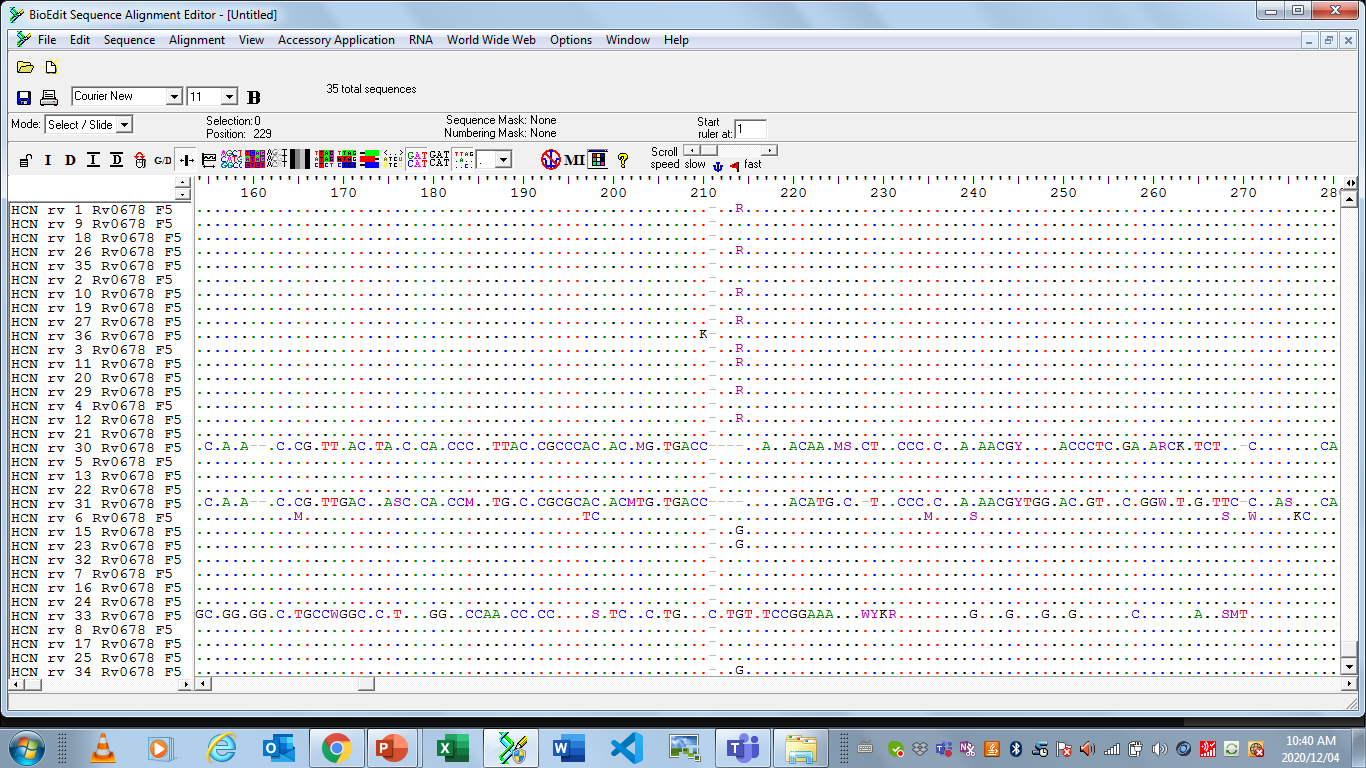

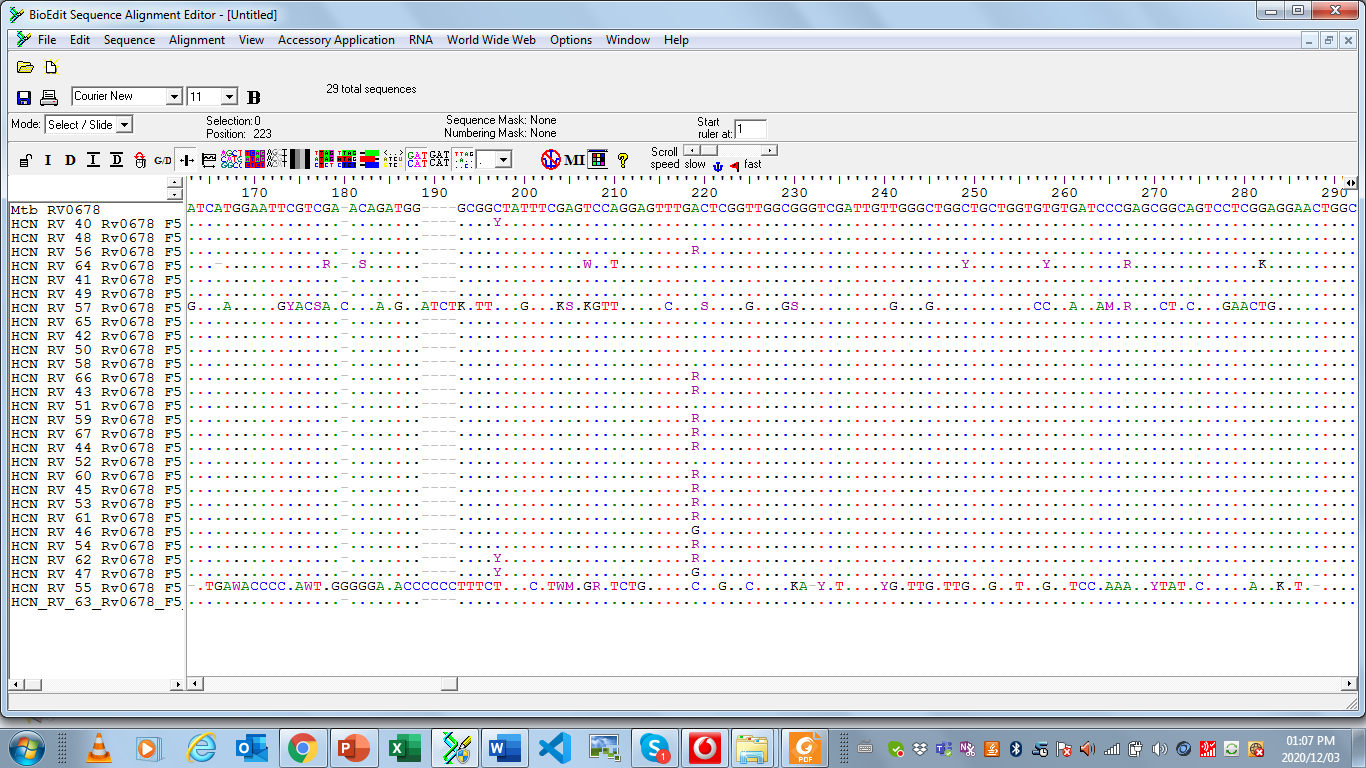


**Figure S3.1: A** Screening of 33 colonies for the *atpE* mutation. The top part shows a first set of 14 colonies, of which one (C11) contained a mixed population of both wild-type and mutated alleles at position 196 (indicated with R). The bottom part shows a second set of 19 colonies, of which one (C14) had a pure mutant population. The C14 colony was selected for further analyses. **B** Screening of 62 sucrose sensitive colonies for the *Rv0678* mutation compared to the H37Rv reference genome (*Rv0678* gene). Seventeen single colonies were mutated, of which 12 colonies carried mixed populations of both wild type and mutant alleles (R at position 214 on the alignment). Five colonies had pure mutant populations. C15 and C46 were selected for further analyses.

**A**
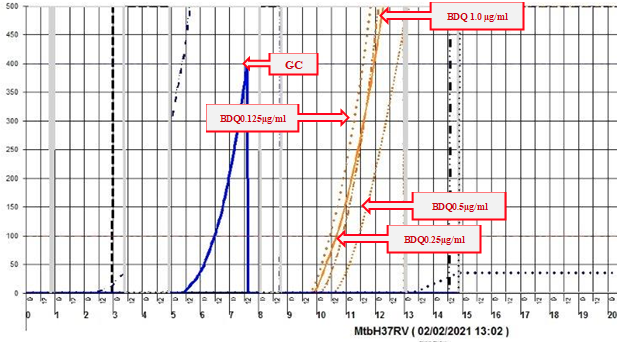
 **B**
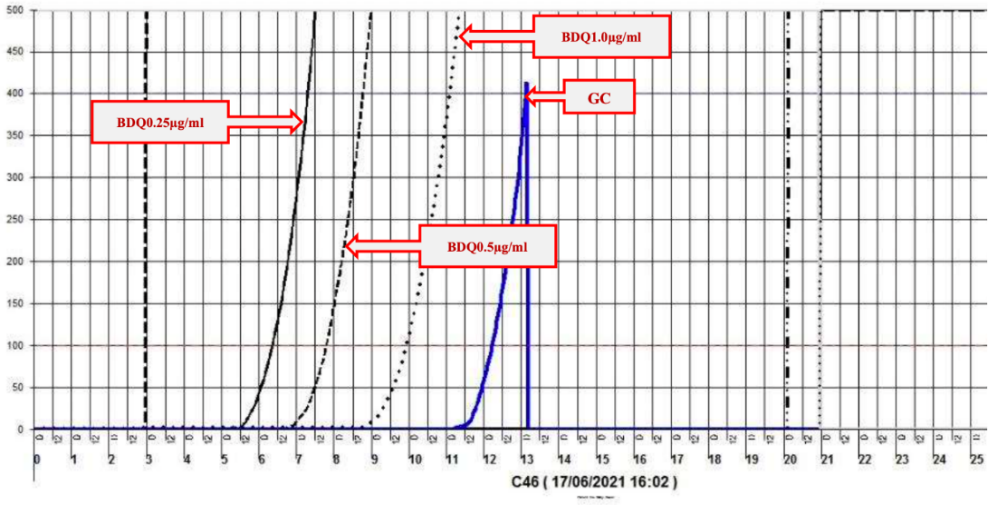


**C**
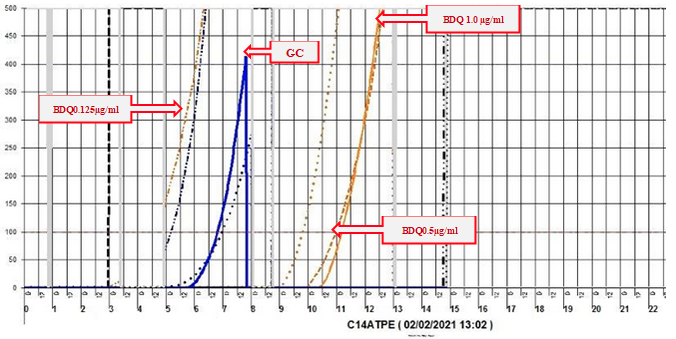
 **D**
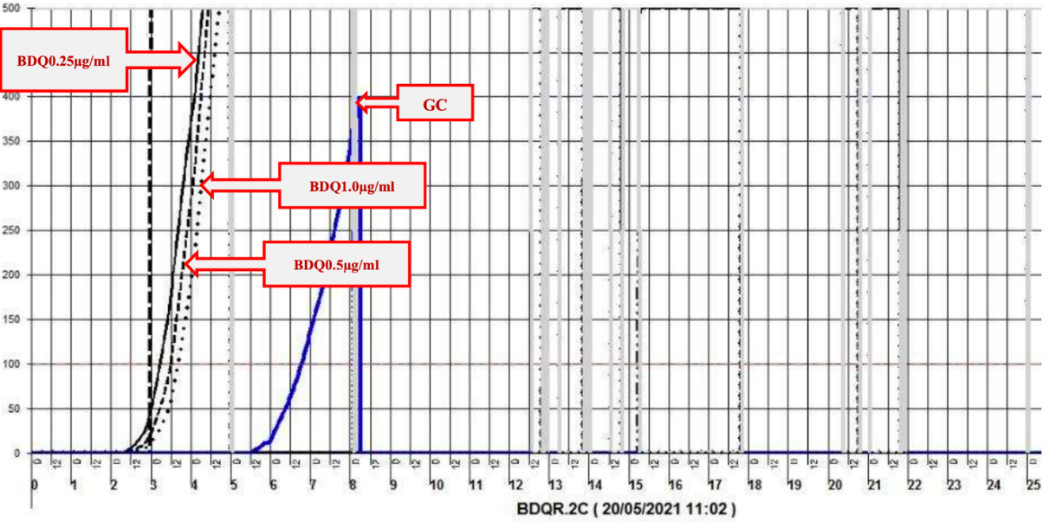


**Figure S3.2: MGIT MIC determinations: A** *Mycobacterium. tuberculosis* H37Rv MGIT MIC determination for BDQ (1.0μg/ml, 0.5μg/ml,0.25μg/ml, 0.125μg/ml), CFZ and drug free growth control (GC). **B** *Mycobacterium. tuberculosis* *Rv0678* Thr33Ala mutant strain MGIT MIC determination for BDQ (1.0μg/ml, 0.5μg/ml, 0.25μg/ml, 0.125μg/ml) and drug free growth control (GC). **C.** *Mycobacterium. tuberculosis* *atpE* Ile66Val MGIT MIC determination for BDQ (1.0μg/ml, 0.5μg/ml, 0.25μg/ml, 0.125μg/ml), CFZ, and drug free growth control (GC). **D** *Mycobacterium. tuberculosis* *atpE* Ala63Pro clinical reference strain MGIT MIC determination for BDQ (1.0μg/ml, 0.5μg/ml, 0.25μg/ml), and drug free growth control (GC).
